# Public Awareness and Use of German Physician Ratings Websites: Cross-Sectional Survey of Four North German Cities

**DOI:** 10.2196/jmir.7581

**Published:** 2017-11-09

**Authors:** Stuart McLennan, Daniel Strech, Andrea Meyer, Hannes Kahrass

**Affiliations:** ^1^ Institute for Biomedical Ethics Universität Basel Basel Switzerland; ^2^ Institute for History, Ethics and Philosophy of Medicine Hannover Medical School Hannover Germany; ^3^ Division of Clinical Psychology and Epidemiology Department of Psychology Universität Basel Basel Switzerland

**Keywords:** physician rating websites, patient satisfaction

## Abstract

**Background:**

Physician rating websites (PRWs) allow patients to rate, comment, and discuss physicians’ quality. The ability of PRWs to influence patient decision making and health care quality is dependent, in part, on sufficient awareness and usage of PRWs. However, previous studies have found relatively low levels of awareness and usage of PRWs, which has raised concerns about the representativeness and validity of information on PRWs.

**Objective:**

The objectives of this study were to examine (1) participants’ awareness, use, and contribution of ratings on PRWs and how this compares with other rating websites; (2) factors that predict awareness, use, and contribution of ratings on PRWs; and (3) participants’ attitudes toward PRWs in relation to selecting a physician.

**Methods:**

A mailed cross-sectional survey was sent to a random sample (N=1542) from four North German cities (Nordhorn, Hildesheim, Bremen, and Hamburg) between April and July 2016. Survey questions explored respondents’ awareness, use, and contribution of ratings on rating websites for service (physicians, hospitals, and hotels and restaurants) and products (media and technical) in general and the role of PRWs when searching for a new physician.

**Results:**

A total of 280 completed surveys were returned (280/1542, 18.16% response rate), with the following findings: (1) Overall, 72.5% (200/276) of respondents were aware of PRWs. Of the respondents who were aware of PRWs, 43.6% (86/197) had used PRWs. Of the respondents who had used PRWs, 23% (19/83) had rated physicians at least once. Awareness, use, and contribution of ratings on PRWs were significantly lower in comparison with all other rating websites, except for hospital rating websites. (2) Except for the impact of responders’ gender and marital status on the awareness of PRWs and responders’ age on the use of PRWs, no other predictors had a relevant impact. (3) Whereas 31.8% (85/267) of the respondents reported that PRWs were a very important or somewhat important information source when searching for a new physician, respondents significantly more often reported that family, friends and colleagues (259/277, 93.5%), other physicians (219/274, 79.9%), and practice websites (108/266, 40.6%) were important information sources.

**Conclusions:**

Whereas awareness of German PRWs appears to have substantially increased, the use of PRWs and contribution of ratings remains relatively low. Further research is needed to examine the reasons why only a few patients are rating physicians. However, given the information inequality between provider and consumer will always be higher for consumers using the services of physicians, it is possible that people will always rely more on interpersonal recommendations than impersonal public information before selecting a physician.

## Introduction

When searching for a new physician, patients typically want to find a physician who is a *good* physician—clinically expert and, at the same time, interested in them, kind, courteous, empathetic, and caring [1]. Yet partly because of a lack of publicly available health care quality information, members of the public have traditionally had few ways of knowing who the *good* physicians are [2]. However, just as the public has used rating websites to find out information about the quality of other products and services, they are also increasingly using physician rating websites (PRWs) to obtain information about physicians, which allow the public to anonymously rate, comment, and discuss physicians’ quality as a source of information for others [3-6]. In contrast to *top-down* public reporting approaches (Web 1.0), which only allow passive viewing of content (eg, the public reporting of health service performance using predetermined standards), PRWs represent a *bottom-up* public reporting approach (Web 2.0), which allows users to also generate content in the form of ratings and comments on physicians’ quality as a form of electronic word of mouth [7-10].

Typically grounded in the assumptions of a theoretical consumer choice model [11], PRWs are a type of *public reporting activity* and have 2 key aims: (1) influencing patient decision making by increasing the chance that those patients who obtain information will choose better quality organizations or individuals [11-12] and (2) driving quality improvement by identifying aspects of care needing improvement so that changes can be made in practice [11-12]. A number of empirical studies indicate that PRWs are having some success in achieving these goals. For instance, a cross-sectional survey conducted in 2013 with a Web-based panel in Germany found that PRWs seem to have a meaningful influence (positive and negative) on choosing a physician [13]. A number of studies have also indicated that there is an association between PRWs and the quality of care. A cross-sectional survey of 2360 German physicians and other health care providers in 2015 reported that more than half of the responding providers used Web-based ratings to derive measures to improve patient care [14]. Furthermore, other studies have found a correlation between PRWs’ ratings and objective measures of quality [15-18]. However, a number of shortcomings of PRWs have also been identified in the literature [19]. These include rating being anonymous, and therefore, not risk-adjusted and vulnerable to fraud, and a low number of ratings that are overwhelmingly positive [19]. This has raised concerns about the representativeness, validity, and usefulness of information on PRWs [19]. Indeed, without higher number of ratings, PRWs will continue to have limited value.

The utilization of the comparative quality information by health consumers depends on a range of factors, but at the most basic level, it requires consumers to first be aware of it [20], and it has been suggested that one reason for the low usage of PRWs might be that patients are still unaware of these websites [5]. However, 2 US studies published in 2014 by Hanauer et al found that whereas 74% of parents and 65% of adults in a nationally representative sample of the US population were aware of PRWs, only 28% of parents and 23% of adults had used PRWs [21,22]. Indeed, the level of PRW awareness reported by these 2 US studies were substantially higher than those found in previous German and English studies [9,13,23], however, the level of usage was comparable with the German studies [9,13]. This suggests that even if awareness of PRWs increases, there are more important factors behind the low level of PRW usage. For example, a Web-based survey of 1006 randomly selected German patients conducted in 2012 found that younger people, women, the highly educated, and people with chronic diseases were more likely to use PRWs [9]. However, regression analyses found that sociodemographic variables and health status alone did not predict PRW usage, but once psychographic variables and information-seeking behavior variables were added, it was found that higher education, poorer health status, higher digital literacy, lower importance of family and pharmacist for health-related information, higher trust in information on PRWs, and higher appraisal of usefulness of PRWs served as significant predictors for PRW usage [9].

With the most recent previous German studies regarding PRW awareness and usage having been conducted in 2012 and 2013 [9,13], there is a need to reexamine this issue to determine where future efforts to increase the level of PRW usage and ratings of physicians should be focused. Whereas both of the most recent German studies have drawn their samples from Web-based panels, this study will take a different approach to data collection and use a random sample of the general public, which should reveal a more generalizable view of the average population compared with panel data. This study aims to examine (1) the level of awareness and usage of PRWs among the general public and how this compares with other rating websites; (2) factors that predict awareness, use, and contribution of ratings on PRWs; and (3) attitudes toward PRWs in relation to selecting a physician.

## Methods

This study was approved by Hannover Medical School’s Research Ethics Committee on January 12, 2016. All participants signed an informed consent form.

### Survey Implementation

A mailed survey was conducted between April and July 2016. A random sample was obtained from the Registry offices of four North German cities of various sizes, under paragraph 34 of the Federal Registry Act (Bundesmeldegesetz) that allows registration authorities to transfer data to other public bodies if certain criteria are met. Inclusion criteria for the random samples were that the person’s place of residency was Nordhorn (53,285 residents, valid December 31, 2015), Hildesheim (101,667 residents, valid December 31, 2015), Bremen (556,326, valid December 1, 2015), or Hamburg (1,787,408 residents, valid December 31, 2015) and that the person was aged between 18 and 85 years. These cities were selected to enable participants from different sized cities to be recruited (small or rural area, medium city, large city, and extra-large city). To ensure participants’ confidentiality and to allow for reminders to be sent to nonresponders, a unique identifier code was assigned to each participant from the random sample before data collection. The document with participants’ identifying information and unique ID was accessible only to the study team, password protected, and stored separately from data documents. Surveys with participants’ unique IDs on them were mailed to a total of 1600 residents in Nordhorn (n=400), Hildesheim (n=400), Bremen (n=400), and Hamburg (n=400). Surveys were sent to residents of Nordhorn, Hildesheim, and Hamburg in the first week of April 2016, with a reminder sent to all nonrespondents 3 weeks later. Due to a late response from the Bremen Registry Office, surveys were sent to residents of Bremen in the first week of June 2016, with a reminder sent to all nonrespondents 3 weeks later. No incentives to participate in the study were provided. Fifty-eight surveys, which were returned because of out-of-date addresses or because participants had died or have a severe disability and are unable to read and write, were excluded from the study, leaving a total of 1542 surveys.

### Survey Contents

Survey questions were primarily adapted from previous surveys conducted in Germany [9,12] and the United States [21,22]. The survey was pilot-tested with a convenience sample of 5 lay people using “think aloud cognitive interview” to ensure clarity and item comprehension [24]. Survey questions explored respondents’ awareness, use, and contribution of ratings on rating websites for service (physicians, hospitals, and hotels and restaurants) and products (media and technical) in general and the role of PRWs when searching for a new physician. Questions concerning the importance of different information sources when searching for a physician, the usefulness of the information in PRWs, and how strongly this information influenced the decision regarding finding a physician used a 4-point Likert scale (eg, from “very important” to “not at all important”). Demographic questions asked for respondents’ age, gender, marital status, education, whether they had previously been employed in health care, type of health insurance, whether they suffer from a chronic illness, and how often they had moved place of residence in the last 10 years (see Multimedia Appendix 1).

### Data Analysis

For the questions concerning the awareness, use, and personal contribution of a rating on rating websites, there exists a cascade of questions where an inconsistent answer pattern could arise if a respondent answered “yes” to a question following a question they had answered “no” to. This inconsistency was solved by only including the first answer. Descriptive statistics included medians and means for continuous variables and percentages for categorical variables. Questions that used 4-point Likert response scales were dichotomized at the midpoint because sample sizes for some cells were often too small to be analyzed. Pearson chi-squared tests were used to analyze awareness, use, and contribution of ratings on rating sites, the role of PWRs when searching for a new physician, characteristics of respondents, and patterns of nonresponse. To test response rates between measures of the same subject (eg, awareness of PRW vs awareness of rating websites for hotels), we used the McNemar test. When comparing the percentages of participants using rating websites between two types of websites (PRWs and another), only those respondents who were aware of both types of websites were included. Similarly, when comparing the percentages of participants contributing a rating on two types of websites (PRWs and another), only those respondents who had used both types of websites were included. To assess potential predictors of the three outcomes—(1) awareness of PRWs, (2) use of PRWs, and (3) previously rated a physician—nine candidate predictors were preselected based on theoretical considerations and previous findings [9,13,21,22], including city, age, gender, marital status, education, previously worked in health care, health insurance, suffers a chronic illness, and number of times place of residence has changed in the last 10 years. We used three different models to test the impact of these predictors. First, we ran univariate logistic regression analyses for each predictor in a separate model, provided regression coefficients are unadjusted for all other predictors. Second, we used multiple logistic regression (MLR) models to test all predictors simultaneously, provided regression coefficients are adjusted for all other predictors in the model. MLRs, however, often suffer from overfitting, especially if the number of predictors is high relative to the number of cases and/or the frequency of the smaller group (in the case of dichotomous outcomes) [25], leading to models with low predictive accuracy when predicting new samples. To avoid such overfitting, we used a variable selection procedure, the least absolute shrinkage and selection operator (lasso), as a third model. In the lasso, model coefficients are deliberately shrunk by implying a penalty term to the binomial likelihood function when fitting the model. As a consequence, these models are somewhat more biased than those obtained from MLR but instead exhibit strongly increased predictive accuracy [26]. Thus, predictors whose coefficients from penalized regression have not been shrunk to 0 are likely to be predictive when replicating the study under consideration. Since the outcome variable was dichotomous, the lasso was based on a logistic regression model. The predictive accuracy of both the MLR and the lasso were determined by cross-validation [27]. We used Cohen kappa, computed between predicted and observed values, and the area under the receiver operating characteristics curves (ROC area) as measures of accuracy. A kappa value of 1 thereby denotes perfect predictive accuracy, whereas a value of 0 denotes random guessing. Accordingly, a value for ROC area of 1 denotes perfect predictive accuracy, whereas a value of 0.5 means random guessing. All predictor variables in the model were standardized before the analysis in any model, except for city and educational level.

## Results

### Characteristics of Respondents

Overall, a total of 280 completed surveys were returned, corresponding to an 18.16% (280/1542) response rate. Seventy-five of the completed surveys came from Nordhorn, 72 from Hildesheim, 62 from Bremen, and 71 from Hamburg. In addition, 169 formal refusals to participate in the study were received, of which 103 provided a reason for nonparticipation. Key reasons given for nonparticipation included no interest in the topic (n=21), no computer and/or Internet (n=10), not aware or do not use PRWs (n=9), health reasons (n=9), time reasons (n=5), and age reasons (n=5). Overall, 15.7% (44/280) of respondents were aged 30 years and less, 29.3% (82/280) were aged between 30 and 50 years, 38.6% (108/280) of respondents were aged between 50 and 70 years, and 16.4% (46/280) were 70 years and older. Furthermore, 55.0% (154/280) of respondents were female, 58.9% (165/280) were married or in a civil partnership, 78.4% (218/278) had never been employed in health care, 81.4% (227/279) had public health insurance, 35.1% (98/279) suffered from a chronic illness, 29.7% (83/279) had changed their place of residence in the last 10 years 1 to 2 times, and 10.0% (28/279) had changed 3 or more times. Nonresponder analysis comparing all responders (n=280) with all nonresponders (n=1320) showed that gender composition did not significantly differ between responders and nonresponders (χ^2^_1_=1.9, *P*=.16), whereas mean age did (responders were older by 3.14 years on average, *t*_1536_=2.68, *P*=.007); however, the effect size was small (*d*=0.18).

### Awareness of Rating Websites

**Figure 1:**
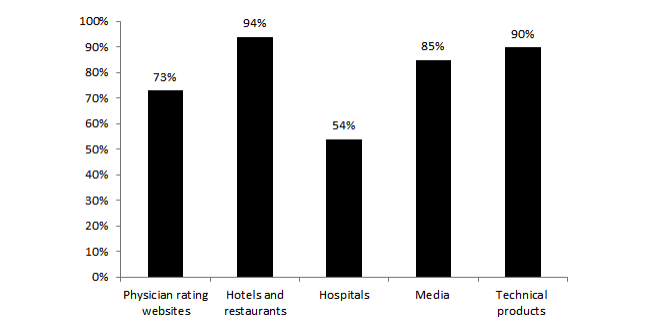
Proportion of respondents who were aware of rating websites.

Overall, 72.5% (200/276) of the respondents were aware of PRWs (see Figure 1; Multimedia Appendix 2 presents the complete results). Respondents’ awareness of PRWs was significantly lower than their awareness of rating websites for hotels and restaurants (χ^2^_1_=52.3, *P* ≤.001), technical products (χ^2^_1_=36.2, *P* ≤.001), and media (χ^2^_1_=18.8, *P* ≤.001), though significantly higher than that for hospitals (χ^2^_1_=33.9, *P* ≤.001). There was also a significant difference between participants’ employment in the health care system and awareness of rating websites for physicians (χ^2^_2_=8.3, *P*=.02; 92% of the participants currently employed in health care were aware of PRWs, 59% of the participants previously employed in health care were aware of PRWs, and 72% of the participants never employed in health care were aware of PRWs).

### Use of Rating Websites

**Figure 2:**
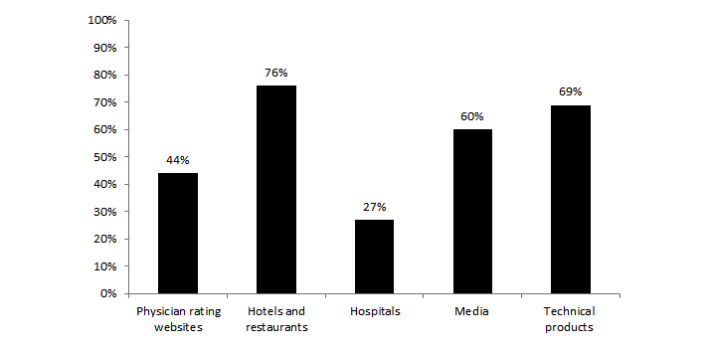
Proportion of respondents who had used rating websites.

Of the respondents who were aware of PRWs, 43.6% (86/197) had used PRWs (see Figure 2; Multimedia Appendix 2 presents the complete results). In comparison with other rating websites, this was significantly lower than the proportion who had used rating websites for hotels and restaurants (χ^2^_1_=44.2, *P≤*.001), technical products (χ^2^_1_=23.2, *P≤*.001), and media (χ^2^_1_=13.0, *P≤*.001), though significantly higher than for hospitals (χ^2^_1_=8.2, *P*=.004). There was also a significant difference between age groups and use of PRWs (χ^2^_3_=10.3, *P*=.02; 58%: 30 years or less, 54%: 30-50 years, 36%: 50-70 years, and 24%: 70 years and above).

### Contributing to Rating Websites

**Figure 3:**
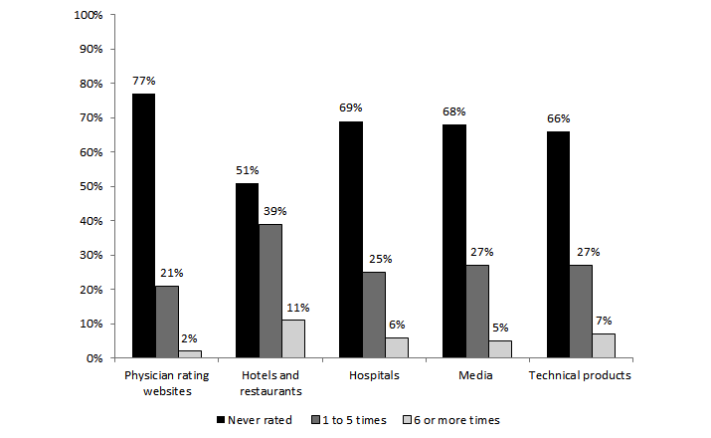
Proportion of respondents who had personally rated on rating websites.

Of the respondents who had used PRWs, 23% (19/83) had rated physicians at least once (see Figure 3; Multimedia Appendix 2 presents the complete results). This value was comparable with the proportion of ratings for the other websites except for hospitals where the respective value of 50% was significantly higher. Statistics comparing the proportions of ratings for physicians with each of the other rating sites were as follows: hotels and restaurants (χ^2^_1_=11.6, *P≤*.001), media (χ^2^_1_=4.8, *P*=.03), technical products (χ^2^_1_=3.5, *P*=.06), and hospitals (χ^2^_1_=0.2, *P*=.65).

### Factors Predicting Awareness and Use of PRWs

Standardized coefficients of all predictors across all 3 outcomes were generally very low, suggesting that except for the impact of responders’ gender (higher for females than for males) and marital status (higher if married or in a civil partnership than if not) on awareness of PRWs and responders’ age on use of PRWs (higher for younger), no other predictors had a relevant impact. Complete results of the tests assessing potential predictors for the outcomes “already aware of PRWs,” “previously used a PRW,” and “previously rated a physician on a PRW” are shown in Multimedia Appendix 3. Results of cross-validation are shown in Multimedia Appendix 4. Those for “rated a physician on a PRW” were below 10%, whereas for the other two outcomes class imbalance was less severe. Predictive accuracy based on cross-validation was low for both the MLR and the lasso. Kappa values varied between 0 and 0.1 and ROC area values between 0.51 and 0.63 depending on the model and the outcome used. The corresponding values based on the sample data where the models were applied were somewhat higher, pointing to the degree of overfit in the MLR.

**Table 1:** Selecting a new physician.

Question		n (%)
**When searching for a new physician, how important were the following information sources? (very or somewhat important)**		
	Family, friends, and colleagues	259 (93.5)
	Other physicians	219 (79.9)
	Practice websites	108 (40.6)
	Physician rating sites	85 (31.8)
	Business directories	51 (19.0)
**Among those who answered “Yes” for used PRWs^a^: How useful did you find the information on physicians rating sites when searching for a physician?**		
	No experience	10 (12)
	Very useful	9 (11)
	Somewhat useful	28 (33)
	Less useful	34 (40)
	Not at all useful	4 (5)
**Among those who answered “Yes” for used PRWs^a^: How strongly did the information on physician rating sites influence your decision regarding a new physician?**		
	No experience	9 (10)
	Very strongly	7 (8)
	Somewhat strongly	22 (26)
	Less strongly	33 (38)
	Not at all	15 (17)

^a^PRWs: physician rating websites.

### Selecting a New Physician

Whereas 31.8% (85/267) of the respondents reported that PRWs were a very important or somewhat important information source when searching for a new physician, they significantly more often reported that other factors were very important or somewhat important information sources, with 93.5% (259/277) endorsing family, friends, and colleagues, 79.9% (219/274) other physicians, and 40.6% (108/266) practice websites (*P* ≤.001 for all three comparisons; see Table 1). There were significant differences between frequency of residency changes and importance placed on PRWs as an information source when searching for a new physician, with 24% considering PRWs as somewhat or very important among those who never moved, 41% among those who moved 1 to 2 times, and 46% among those who moved 3 or more times (χ^2^_2_=10.3, *P*=.006). For those respondents who had previously used PRWs, 44% (37/85) reported that they found the information on PRWs very useful or somewhat useful when searching for a new physician. Similarly, 34% (29/86) of the respondents who had previously used PRWs reported that the information on PRWs very strongly or somewhat strongly influenced their decision regarding a new physician, although this increased to 50% (11/22) among respondents who had previously rated a physician.

## Discussion

### Principal Findings

This study has resulted in three key findings. First, awareness of German PRWs was found to be high (72.5%), though the usage of PRWs (43.6%) and especially the contribution of ratings (23%) remain relatively low. Awareness, use, and contribution of ratings on PRWs were also significantly lower in comparison with all other rating websites, except for hospital rating websites. Second, respondents’ age was the only relevant predictor of use of PRWs, with younger respondents more likely to use PRWs. Third, when selecting a new physician, the importance of factors such as family and friends as information sources were endorsed more frequently than PRWs.

Respondents’ reported awareness of PRWs was substantially higher than the awareness of PRWs reported by previous German studies, which found 29% awareness in 2012 and 32% in 2013 [9,13]; however, it is similar to the 2 US studies published in 2014 by Hanauer et al, which found that 74% of parents and 65% of adults in a nationally representative sample of the US population were aware of PRWs [21,22]. It therefore appears that a lack of PRW awareness is no longer a key barrier to PRW usage (in Germany and the United States) [5]. The apparent increase in awareness of German PRWs may have been influenced by the low response rate, with responders more likely to already be aware of PRWs and the majority of nonresponders said they were not interested in the topic. However, it is also potentially due to large German public health insurers launching their own PRWs. Germany’s largest public health insurer Allgemeine Ortskrankenkasse (AOK) launched the PRW Arzt-Navigator in 2010, which was rolled out nationwide in May 2011. Two other large public health insurers, Techniker Krankenkasse (TK) and BARMER GEK, have also subsequently developed their own PRWs (TK-Ärzteführer and BARMER GEK-Arztnavi, respectively). These PRWs have received media attention that has likely led to a much greater public awareness of PRWs [28].

However, despite this increase in PRW awareness, the percentage of those who had used PRWs (among the responders who were aware of them) was substantially lower than previous German studies. For instance, whereas Emmert et al only reported that 25% of all respondents had used PRWs, their data show that among those who were aware of PRWs, 79% had used PRWs [13]. Similarly, while Terlutter et al only reported 26% of all respondents used PRWs at least once, their data show that among those who were aware of PRWs, 88% had used PRWs [9]. It is noteworthy that these numbers are even higher than the rate of users of the most common rating website in our survey (hotels and restaurants: 76%). The large difference between our study and the previous German studies likely reflects the increase in awareness of German PRWs and the different methodologies used in the 2 studies. When the previous German studies were conducted, most German PRWs were still in a growth phase and awareness of German PRWs was low; furthermore, both the Emmert et al and Terlutter et al studies used Web-based surveys and recruited participants from Web-based panels [9,13]. Our study, on the other hand, has been conducted when German PRWs are in a more mature phase, and awareness of German PRWs is much higher; additionally, our study used a mailed survey and a sample of the general public. Consequently, our study’s findings more likely reflect the distribution between PRWs awareness and usage in the general public, although further research is needed to confirm this. Nevertheless, the 2 US studies by Hanauer et al involving the general public also found similar rates of PRW usage among those aware of PRWs: 38% and 37% [21,22].

Respondents’ age was found to be the only relevant predictor of use of PRWs, with younger respondents more likely to use PRWs. This finding supports previous research [9,29] and the suggestion that the main impact of PRWs will likely come with the next “Facebook” generations, who use the Internet more and are more used to providing ratings on the Internet [30]. The use of PRWs is also likely influenced, in part, by the importance patients place on it as an information source when searching for a new physician. Whereas the vast majority of responders reported that family, friends, and colleagues were a very or somewhat important information source, only one-third of responders considered PRWs an important source. The 2 US studies by Hanauer et al reported that participants placed similar importance on family and friends as an information source (very or somewhat important: 85% and 89%, respectively) but substantially higher importance on PRWs (very or somewhat important: 59% and 62%, respectively) compared with the German participants in our study [21,22]. The large difference placed by US and German participants on the importance of PRWs as an information source when searching for a new physician may be partly because of differences in population mobility in the 2 countries; our study found that the proportion of German participants placing importance on PRWs increased with more changes of residency; however, research suggests that the US population is more mobile than European Union citizens [31]. Population mobility may be a fruitful area for future research to examine in relation to differences between countries in the use of PRWs.

Among the responders who had used PRWs, the percentage of those who had rated a physician was also much lower than previous German studies. For instance, whereas Emmert et al reported that only 11% of all respondents had rated a physician, their data show that among those who had used PRWs, 44% had rated a physician [13]. This higher rate of rating may also reflect the Web-based survey and Web-based panel used by Emmert et al. Previous international studies have found results similar to our study, with both US studies by Hanauer et al reporting that among those who had used PRWs, 23% had rated physicians [21,22]. As PRW users are not compensated for contributing ratings in any way, basic economic theory suggests that ratings were likely to be underprovided [32]. However, it is clear that users of other rating websites are contributing ratings more often. Whereas the lower awareness and usage of PRWs in comparison with other rating websites may remain a factor, there are clearly other factors that are leading patients not to rate their physicians given the low number of people who use PRWs going on to contribute their own rating. Of the responders who had used PRWs, under a third had contributed a rating. Two-thirds of PRW users are therefore effectively *free riders*, who are willing to use PRWs as a source of information but do not contribute ratings. However, without higher number of ratings, PRWs will continue to have limited value for every user. It is therefore not only a matter of reciprocity, but it is in the users’ self-interest to contribute ratings. Many PRW users may not be aware of this, and PRWs may want to consider notifying users of the importance of contributing ratings.

However, it is also useful to consider the reasons why participants’ awareness and usage of PRWs were all significantly lower in comparison with the other non–health care-related rating websites, which is consistent with the results of the 2 Hanauer et al studies [21,22]. In a recently published study by Rothenfluh et al, the choice-making processes of participants using the rating website TripAdvisor to select a hotel and the PRW Jameda to select a physician were explored [33]. Despite involving 2 service goods, major differences between the uses of the 2 rating websites were found. Whereas participants thought that choosing a physician was more important than choosing a hotel, participants spent less time searching for physicians than hotels and found choosing a physician much easier. Four themes were identified as being behind these differences. First, participants used a “trial and error” approach when selecting a physician, deciding only after a visit if they wanted to stay or switch to another, whereas they felt confident in making a definite choice regarding a hotel based on the information on the rating website. Second, participants expressed high trust in the medical profession and perceived that any of the listed physicians would be competent, while their confidence in hotels was lower. Third, although participants felt confident in evaluating the quality of a hotel, they perceived that they had an inability to properly evaluate the skills and abilities of physicians. Finally, participants reported that interpersonal connection, gut feeling, and likeability played a huge role in selecting a physician, whereas the price and offered facilities were more important in relation to hotels [33]. Drawing from the economics literature, Rothenfluh et al distinguished between different types of services goods according to the level of information asymmetry between the provider and consumer and consequently suggested that these 2 service goods (hotels and physicians) cannot be treated equally because of their unequal attributes [33].

These findings suggest that the awareness and usage of PRWs may, in fact, always be lower in comparison with the other non-health care–related rating websites because the information inequality between provider and consumer will always be higher for consumers using the services of physicians (and hospitals) than it is for consumers using other services such as hotels and restaurants, and even more so for consumers buying products such as media and technical products. Whereas changes to the way in which PRWs are currently designed may make them more useful [33], it is possible that people will always rely more on interpersonal recommendations than impersonal public information before selecting a physician, and consequently not use PRWs as often as they would other rating websites. It is therefore perhaps not very surprising that participants endorsed far more frequently the importance of family and friends as information sources than PRWs when selecting a new physician, which is consistent with previous research [21,22].

Nevertheless, there is currently limited research examining the reasons why patients are not rating their physicians on PRWs, and more research is needed regarding this issue to identify barriers that may be addressed. A recently published study by Patel et al explored patients’ views regarding rating general practitioners on PRWs, within the context of other feedback methods available in England [34]. Participants reported that they would not leave feedback on PRWs because of accessibility issues, privacy and security concerns, and because they felt feedback left on a website may be ignored [34]. Hanauer et al also asked participants in their 2012 US study to consider the implications of leaving negative comments about a physician [22]. Participants reported being concerned that their identity could be disclosed (34%) and that the physicians may take action against them for leaving negative comments (26%) [22].

### Limitations

This study has a number of limitations that should be taken into account when interpreting the results. Responder bias may have influenced the results; however, as those who responded to our survey are likely to be generally more interested in the issue, the relatively low use of PRWs and low contribution of ratings should be taken seriously. Additionally, with a response rate of 18.1%, a generalization of the quantitative results to all the inhabitants of the four North German cities is likely not possible. Differences may also exist between other regions in Germany with respect to rating sites. However, the survey was mailed to a random sample of an average population from four North German cities of different sizes. The nonresponder analysis also found no significant difference in gender, and whereas responders were slightly older than nonresponders on average, the effect size was small. One of the reasons nonparticipants provided for not participating in the survey was that they were not aware of PRWs or do not use PRWs. If nonparticipants did not participate simply because they were not aware of PWRs, the average proportion of participants that is aware of PRWs is likely to be overestimated. However, out of the 169 refusals to participate in the study that were received, only 9 of them reported that they did not participate because they are not aware of PRWs or do not use PRWs. Furthermore, as there is a cascade of questions (awareness > use > contribution of ratings), there need not be an overestimation in relation to the use of PRWs and rating of a physician on a PRW at least once, since only participants who are actually aware of PWRs were included for these questions. We therefore do not think that this issue has significantly impacted our results. Responses were self-reported, and therefore we do not know the actual use of PRWs or what PRW participants visited. Additional research involving a larger sample would be desirable.

### Conclusions

This study indicates that awareness of PRWs in Germany has substantially increased in recent years. This is a positive development and suggests that a lack of awareness is no longer a key barrier for PRW usage in Germany. Nevertheless, the level of usage of PRWs remains relatively low, and moving forward, the focus should be on a better understanding of the reasons why patients are not rating their physicians on PRWs, so that barriers that may be addressed can be identified. However, given the fact that the information inequality between provider and consumer will always be higher for consumers using the services of physicians, the awareness and usage of PRWs may, in fact, remain lower in comparison with the other non-health care–related rating websites. While changes to the way in which PRWs are currently designed may make them more useful, it is possible that people will always rely more on interpersonal recommendations than impersonal public information for selecting a physician.

## Appendix

Multimedia Appendix 1 Survey.

Multimedia Appendix 2 Awareness and use of rating websites.

Multimedia Appendix 3 Factors predicting awareness and use of physicians rating websites.

Multimedia Appendix 4 Model accuracy for multiple logistic regression model and the lasso based on 10-fold repeated cross-validation.

## Abbreviations

AOK: Allgemeine Ortskrankenkasse
HiLF: Hochschulinterne Leistungsförderung
MLR: multiple logistic regression
PRWs: Physician rating websites
ROC area: area under the receiver operating characteristics curves
TK: Techniker Krankenkasse
