# Supplementary material for: Public Awareness and Use of German Physician Ratings Websites: Cross-Sectional Survey of Four North German Cities
Source: J Med Internet Res. 2017 Nov 9;19(11):e387. doi: 10.2196/jmir.7581 (PMC5701087; doi:10.2196/jmir.7581)
Supplement: Multimedia Appendix 1 [file jmir_v19i11e387_app1.pdf]

Multimedia Appendix 1: Survey

## Bewertungsportale im Internet
## Rating Websites on the Internet

Im Internet hat jeder die Möglichkeit, Produkte oder Dienstleistungen zu bewerten und Erfahrungsberichte hierzu abzugeben. Andere Internetnutzer können die Informationen auf den Bewertungsportalen abrufen und zur Orientierung bei ihrer Suche nach einem Produkt oder nach einer Dienstleistung verwenden. Im Folgenden möchten wir Ihnen einige Fragen zu Ihren Erfahrungen mit Bewertungsportalen stellen. Bitte beantworten Sie den Fragebogen auch dann, wenn Sie noch keine eigenen Erfahrungen gemacht haben.

On the Internet, everyone has the opportunity to rate products or services and submit their experiences. Other Internet users can access the information on the rating portals and use them to orient their search for a product or service. In the following we would like to ask you some questions about your experiences with evaluation portals. Please answer the questionnaire even if you have not had your own experiences.

| **1. War Ihnen bereits vor unserer Befragung bekannt, dass es Webseiten gibt, auf denen man folgende Produkte oder Dienstleistungen bewerten kann?<br>Did you already know before our survey that there are websites where you can evaluate the following products or services?** | | |
|---|---|---|
| Physicians | ☐ Yes | ☐ No |
| Hotels or Restaurants | ☐ Yes | ☐ No |
| Hospitals | ☐ Yes | ☐ No |
| Media (e.g. films or books) | ☐ Yes | ☐ No |
| Technical Products (e.g. mobile phones or vacuum cleaners) | ☐ Yes | ☐ No |

| **2. Haben Sie schon einmal Bewertungen zu einem der folgenden Produkte oder Dienstleistungen angeschaut?<br>Have you ever looked at ratings for any of the following products or services?** | | |
|---|---|---|
| Physicians | ☐ Yes | ☐ No |
| Hotels or Restaurants | ☐ Yes | ☐ No |
| Hospitals | ☐ Yes | ☐ No |
| Media (e.g. films or books) | ☐ Yes | ☐ No |
| Technical Products (e.g. mobile phones or vacuum cleaners) | ☐ Yes | ☐ No |

**3. Wie oft haben Sie selbst die folgenden Produkte oder Dienstleistungen bewertet?**
**How often have you personally rated the following products or services?**

| | | | |
|---|---|---|---|
| Physicians | ☐ Keinmal | ☐ 1-5 | ☐ 6 oder mehr |
| Hotels or Restaurants | ☐ Keinmal | ☐ 1-5 | ☐ 6 oder mehr |
| Hospitals | ☐ Keinmal | ☐ 1-5 | ☐ 6 oder mehr |
| Media (e.g. films or books) | ☐ Keinmal | ☐ 1-5 | ☐ 6 oder mehr |
| Technical Products (e.g. mobile phones or vacuum cleaners) | ☐ Keinmal | ☐ 1-5 | ☐ 6 oder mehr |

## Auswahl eines neuen Arztes
## Selecting a New Physician

In den folgenden Fragen geht es um die Rolle von Arztbewertungsportalen bei der Suche nach einem neuen Arzt oder einer neuen Ärztin.

The following questions are about the role of physician rating websites in the search for a new physician.

**4. Bei der Suche nach einem neuen Arzt oder einer neuen Ärztin, wie wichtig sind Ihnen die folgenden Informationsquellen?**

**When searching for a new physician, how important are the following information sources to you?**

| | Very importan t | Somewha t important | Less importan t | Not at all importan t |
|---|---|---|---|---|
| Other physicians | ☐ | ☐ | ☐ | ☐ |
| Physician rating websites | ☐ | ☐ | ☐ | ☐ |
| Business directories | ☐ | ☐ | ☐ | ☐ |
| Family, friends or colleagues | ☐ | ☐ | ☐ | ☐ |
| Practice websites | ☐ | ☐ | ☐ | ☐ |
| Other and namely: | ☐ | ☐ | ☐ | ☐ |

**5. Wie nützlich fanden Sie die Informationen auf Arztbewertungsportalen für die Arztsuche?**

**How useful did you find the information on the physician rating websites for the physician search?**

| | | | | |
|---|---|---|---|---|
| ☐ No experience | ☐ Very useful | ☐ Somewhat useful | ☐ Less useful | ☐ Not at all |

| **6. Wie stark haben die Informationen auf Arztbewertungsportalen Sie in einer konkreten Entscheidung bei der Arztsuche beeinflusst?** | | | | |
|---|---|---|---|---|
| ☐ No experience | ☐ Very strongly | ☐ Somewhat strongly | ☐ Less strongly | ☐ Not at all |

| **7. Wenn Sie selbst noch keine Bewertung abgegeben haben, nennen Sie uns bitte Gründe dafür.** |
|---|
| If you have not yet personally given a rating, please let us know the reasons. |

## Angaben zu Ihrer Person
## Personal details

| **8. In welchem Jahr wurden Sie geboren?**<br>**In what year were you born?** | **9. Ihr Geschlecht**<br>**Your gender** | | |
|---|---|---|---|
| 19 __ __ | ☐ Male | ☐ Female | ☐ Others |

| **10. Ihr Familienstand**<br>**Martial Status** |
|---|
| ☐ Married / Registered Life Partnership, living together |
| ☐ Married / Registered partner, living separately |
| ☐ Single |
| ☐ Divorced |
| ☐ Widowed |

**11. Welchen höchsten Schulabschluss haben Sie?**
**What is your highest school leaving certificate?**

- ☐ Still go to school
- ☐ Left school without a certificate
- ☐ Primary school certificate
- ☐ Junior high school certificate (Middle Maturity)
- ☐ Polytechnic secondary school of the GDR
- ☐ Technical university entrance certificate
- ☐ University entrance certificate

☐ Another school leaving certificate, namely

**12. Welche Ausbildungsabschlüsse haben Sie? Welche Punkte auf dieser Liste treffen auf Sie zu?** Hier können Sie mehrere Antworten ankreuzen.
**What qualifications do you have?** Here you can select several answers.

☐ Still training / studying
☐ No professional qualification and currently not in vocational training
☐ Vocational company training completed (teaching)
☐ Vocational school training completed
☐ Completion of a
☐ Bachelor at a (technical) university
☐ Techinical univeraity degree (e.g. Diploma, Master)
☐ University degree (e.g. Diploma, Master, State examination)
☐ Doctorate
☐ Another qualification,namely ____________________

**13. Haben Sie jemals eine Tätigkeit im Gesundheitswesen ausgeübt?**
**Have you ever wored in healthcare?**

| | | |
|---|---|---|
| ☐ Yes, previously | ☐ Yes, currently | ☐ No, never |

**14. Bei welcher Krankenversicherung sind Sie versichert?**
**What kind of health insurance do you have?**

☐ Private health insurance (fully insured)
☐ Statutory health insurance (e.g. AOK, BKK), and namely: ____________________
☐ Other

**15. Leiden Sie an einer chronischen Erkrankung, derentwegen Sie regelmäßig einen Arzt aufsuchen?**
**Do you suffer from a chronic illness for which you regularly visit a physician?**

| | |
|---|---|
| ☐ Yes | ☐ No |

**16. Wie oft haben sie in den letzten 10 Jahren den Wohnort gewechselt?**
**How often in the last 10 years have you changed your place of residence?**

| | | |
|---|---|---|
| ☐ Never | ☐ 1-2 | ☐ 3 und mehr |
