# Supplementary material for: Public Awareness and Use of German Physician Ratings Websites: Cross-Sectional Survey of Four North German Cities
Source: J Med Internet Res. 2017 Nov 9;19(11):e387. doi: 10.2196/jmir.7581 (PMC5701087; doi:10.2196/jmir.7581)
Supplement: Multimedia Appendix 2 [file jmir_v19i11e387_app2.pdf]

Multimedia Appendix 2: Awareness and use of rating websites

**Type of Rating Website**	**Were you already aware that there are websites that allow people to rate the following products or services?**	**Among those who answered "Yes" for awareness: Have you ever used rating websites for any of the following products or services?**	**Among those who answered "Yes" for using rating websites: How often have you personally rated the following products or services?**		
	Yes (%)	Yes (%)	Never (%)	1-5 (%)	6 or more (%)
Physicians	200/276 (72.5)	86/197 (43.6)	64/83 (77)	17/83 (21)	2/83 (2)
Hotels and restaurants	262/279 (93.9)	198/262 (75.6)	97/191 (50.8)	74/191 (38.7)	20/191 (10.5)
Hospitals	148/273 (54.2)	39/146 (26.7)	25/36 (69)	9/36 (25)	2/36 (6)
Media	234/274 (85.4)	137/229 (59.8)	92/136 (67.6)	37/136 (27.2)	7/136 (5.1)
Technical products	248/275 (90.2)	168/245 (68.6)	109/165 (66.1)	45/165 (27.2)	11/165 (6.6)
