# Supplementary material for: Public Awareness and Use of German Physician Ratings Websites: Cross-Sectional Survey of Four North German Cities
Source: J Med Internet Res. 2017 Nov 9;19(11):e387. doi: 10.2196/jmir.7581 (PMC5701087; doi:10.2196/jmir.7581)
Supplement: Multimedia Appendix 3 [file jmir_v19i11e387_app3.pdf]

Multimedia Appendix 3: Factors Predicting Awareness and Use of Physicians Rating Websites

Predictor	Awareness of PRWs[a]			Used PRWs			Rated a physician on a PRW		
	Uni[b]	Multi[c]	Lasso[d]	Uni	Multi	Lasso	Uni	Multi	Lasso
Age	–0.18 (0.14)	–0.29 (0.20)	–0.25	–0.40 (0.15)**	–0.65 (0.24)**	–0.62	0.21 (0.27)	0.21 (0.42)	0.21
Sex	0.36 (0.14)	–0.38 (0.15)*	0.36	0.18 (0.15)*	0.08 (0.17)	0.08	–0.36 (0.26)	–0.44 (0.32)	–0.44
Marital status	0.17 (0.14)	0.37 (0.17)*	0.32	–0.13 (0.15)	0.10 (0.18)	0.08	0.27 (0.29)	0.17 (0.35)	0.17
Previously worked in healthcare	0.03 (0.14)	–0.06 (0.16)	–0.03	0.07 (0.15)	0.05 (0.16)	0.04	0.12 (0.26)	0.66 (0.36)	0.65
Health care insurance	–0.03 (0.14)	–0.01 (0.15)	0.00	0.38 (0.17)*	0.40 (0.19)*	0.39	–0.22 (0.23)	–0.33 (0.31)	–0.33
Chronic illness	0.12 (0.14)	0.01 (0.15)	0.00	–0.10 (0.15)	–0.35 (0.17)*	–0.33	–0.22 (0.26)	–0.55 (0.36)	0.55
Residence changed in last 10 years	0.11 (0.14)	0.09 (0.18)	0.06	0.35 (0.15)*	0.06 (0.19)	0.06	0.20 (0.26)	0.63 (0.41)	0.63

[a] * p<.05; ** p<.01; *** p<.001

[b] Univariate Model

[c] Multivariate Model

[d] Lasso Model - In the lasso, model coefficients are deliberately shrunk by implying a penalty term to the binomial likelihood function when fitting the model. Predictors whose coefficients from penalized regression have not been shrunk to zero are likely to be predictive when replicating the study under consideration. Coefficients are standardized and thus denote the increase in the log-odds of the dichotomous outcome (awareness, use, or rating of PRWs) for a change in the predictor by one standard deviation
