# Supplementary material for: Public Awareness and Use of German Physician Ratings Websites: Cross-Sectional Survey of Four North German Cities
Source: J Med Internet Res. 2017 Nov 9;19(11):e387. doi: 10.2196/jmir.7581 (PMC5701087; doi:10.2196/jmir.7581)
Supplement: Multimedia Appendix 4 [file jmir_v19i11e387_app4.pdf]

Multimedia Appendix 4: Model accuracy for multiple logistic regression model and the lasso based on 10-fold repeated cross-validation.

**Type of cross-validation**	**Awareness of PRWs (n=266)**	**Use of PRWs (n=192)**	**Rated a physician on a PRW (N=80)**
Base rate (proportion of cases)	0.73	0.42	.23
MLR Model[a], own data			
Kappa [b]	0.12	0.19	0.13
ROC area[c]	0.69	0.70	0.81
MLR Model, cross-validated			
Kappa	0.04	0.08	–0.08
ROC area	0.59	0.60	0.57
Lasso Model[d], cross-validated			
Kappa	0.05	0.10	–0.07
ROC area	0.59	0.60	0.57

[a] Multiple logistic regression

[b] Cohen’s kappa

[c] Receiver operating characteristics curves (ROC)

d Least absolute shrinkage and selection operator (lasso)
